# Assessing the use of ASAP‐MS for Screening a UK Pub for Illicit Drug Substances

**DOI:** 10.1002/jms.70121

**Published:** 2026-10-12

**Authors:** Andriana Michailidis, Simone Mathias, Eloise Edgar‐Gibson, David Douce, Victoria Starkie, Melanie Bailey, Patrick Sears

**Affiliations:** ^1^ School of Chemistry and Chemical Engineering University of Surrey Guildford UK; ^2^ Waters Corporation Wilmslow UK; ^3^ The Department of Infectious Diseases King's College London London UK

## Abstract

Illicit drug use in social environments, such as pubs and bars, has become an increasingly significant issue for public health and safety. These venues often provide a setting where individuals may engage in recreational drug use, resulting in their residual presence on high‐contact surfaces. This study aims to investigate the presence of illicit drugs in a pub within the United Kingdom, focusing on common touchpoints such as the tables, bar and men's/women's bathrooms. Over 400 surface swabs were collected across 3 weeks, with the objective of identifying traces of illicit substances commonly associated with recreational use. This study additionally explored the potential of atmospheric solids analysis probe mass spectrometry (ASAP‐MS) as a screening tool for the on‐site detection of cocaine, ketamine, amphetamine, diazepam and tetrahydrocannabinol (THC). Previous research has demonstrated that ASAP‐MS is an effective method for detecting illicit drugs with minimal sample preparation. An untargeted screening method was developed for direct swab analysis via glass capillary rod, whereas a combined untargeted and targeted approach was used for solvent‐extracted samples. For confirmatory testing, liquid chromatography mass spectrometry (LC–MS) determined the effectiveness of each ASAP‐MS analysis. Results indicated that the targeted method with minimal sample preparation detected trace amounts of illicit drugs that were previously undetected using the initial screening method. However, the untargeted method, with direct sample analysis, showed potential for detecting larger quantities, particularly cocaine. Overall, the findings revealed drug residues at all touchpoints within the pub, particularly in the bathrooms which contained traces of cocaine in all samples.

## 1 Introduction

According to the Office of National Statistics (ONS), approximately 3.1 million people (between the ages of 16–59) within the UK stated that they had used illegal drugs in the year ending March 2023 [1]. This included around 2.5 million cannabis users and 1.1 million Class A drug users. However, these statistics are likely to be an underestimation of the number of adults who have used drugs in the UK if it's based on self‐reported data. The public health implications of illicit drug use relate to both poisoning from drug misuse in addition to drug‐related mental and behavioural disorders, of which during the 2022/2023 period, the latter was responsible for more hospital admissions for individuals aged between 16 and 44 [2]. Patterns of drug use show a temporal trend, with use more likely to occur during the weekend. Research has shown that illicit drugs are detected at higher concentrations within wastewater samples collected from Saturday to Monday [3]. Nightlife venues such as bars, raves and nightclubs have long been associated with illicit drug consumption, where evidence of cannabis, ecstasy, opioids, prescription medication, cocaine and methamphetamine has been observed [4]. In addition, illicit drug consumption among young adults is more prevalent in those who frequent pubs, where cocaine usage has been found to be twice as high for those who attend pubs less frequently.

The prevalence and circulation of illicit drugs have raised concern in relation to the background contamination levels present in various environments. The movement of illicit drugs through handling, improper disposal and their presence within the atmosphere poses a significant risk to public health and safety [5, 6]. Surface transfer via commonly touched objects not only raises public health concerns, but potential implications in illicit drug testing. Research investigating the presence of cocaine on various surfaces found that 35 out of 45 tested surfaces were positive for cocaine. Test surfaces included petrol pumps, bank machines and door handles, which all resulted in a 100% positive response whereas shopping carts and academic building entrances displayed a 70% and 30% positive response, respectively [7]. Despite the high number of positive responses, detection of illicit drugs on commonly handled surfaces, particularly those that are not often cleaned, may be expected. However, research has also shown that detection of illicit drugs in controlled, regularly cleaned environments has been possible. Sisco et al. investigated surfaces within a forensic laboratory, where wipe samples were collected from surfaces across several units for the screening of illicit drugs. Despite the implementation of regular cleaning routines by staff, illicit drug traces were detected throughout the laboratory, with the highest surface concentrations found on weighing balances, where cocaine was present in 95% of the recovered samples [8].

Mass spectrometry (MS) is a useful technique which can be used for the identification and quantification of illicit drugs. It is typically coupled with a chromatographic technique such as gas chromatography (GC) or liquid chromatography (LC). Most published methods utilising GC–MS or LC–MS for the detection of drugs centre around clinical and toxicological applications, where testing is typically carried out using blood, urine and saliva [9, 10, 11, 12]. These matrices require sample preparation before analysis to limit matrix effects such as ion enhancement or suppression. These instruments are associated with high costs due to the initial purchase, ongoing maintenance and the requirement for a trained analyst to prepare and analyse samples. Due to the size, solvents and gas requirements of these instruments they are often not suitable for use outside of a laboratory environment, making them unlikely to be useful for on‐site screening scenarios.

Key requirements for on‐site drug screening include speed and portability, with most drug‐checking corporations opting for Fourier transform infrared (FTIR) and Raman spectroscopy [13]. Despite these instruments being portable, non‐destructive and fast, analysis of mixed and low concentration samples remains a challenge [14]. Ambient ionisation mass spectrometry (AIMS) allows for the direct analysis of samples with minimal sample preparation, eliminating chromatographic separation. When coupled with small footprint mass spectrometers, these techniques are better suited for on‐site analysis compared to LC–MS and GC–MS due to their ease of use and rapid analysis time.

Ambient ionisation was first introduced into the literature with desorption electrospray ionisation (DESI) and direct analysis in real time (DART) in the early 2000s and has since evolved into a variety of techniques with numerous applications in forensics, biomedicine, environmental and food science [15, 16, 17]. One technique is atmospheric pressure solids analysis probe (ASAP) which was developed by McEwen et al. through modifying an atmospheric pressure chemical ionisation (APCI) probe to accommodate a glass capillary rod [18]. Recent studies have found that ASAP in combination with either high‐resolution or single quadrupole mass spectrometers can be a useful tool in forensic analysis for the rapid detection of illicit substances [19, 20]. A recent investigation evaluated portable MS with ASAP for the rapid identification of bulk drug seizures in a deployable setting, conducting an untargeted screening of 50 seized drugs and finding that 49 of the analysed samples' constituents were in full agreement with a confirmatory analysis [21]. Similarly, Frinculescu et al. used similar methods for the on‐site identification of drugs found in Amnesty bins at UK festivals and found that 93% (*n* = 94) of all responses agreed with an established GC–MS method [22].

Through the handling or consumption of illicit substances, traces can be left behind on commonly handled surfaces especially in areas where cleaning practices range from infrequent to routine. In both laboratory settings and public venues, the transfer and contamination of illicit drugs is widespread. This can pose health and safety risks not only to unsuspecting patrons, but also to individuals working in these environments. The need for techniques that are rapid, selective, portable and easy to operate is ongoing and in this work, we explore the feasibility of using ambient mass spectrometry to address this challenge. The creation of realistic standards to reflect real‐world environments can be challenging in a laboratory. By depositing fixed volumes of standards onto surfaces can result in the coffee ring effect and material adhering the surface rather than loose particulates that are organically distributed. To overcome this, a portable mass spectrometer with an ASAP system is tested as a screening device for the detection of illicit drugs present on various surfaces within a pub in the United Kingdom. Samples were collected by dry‐swabbing various surfaces in the pub. The swabs were initially analysed without sample preparation using an untargeted mass spectrometry method. A solvent extraction was then performed on each swab, followed by analysis using a targeted and untargeted method. All solvent extracted samples were additionally analysed using LC–MS for confirmatory testing.

## 2 Experimental

### 2.1 Materials

Ultra LC methanol (MeOH), water and acetonitrile (ACN) were purchased from ROMIL (Cambridge, UK). Formic acid was obtained from Fisher Scientific (Leicestershire, UK). Ammonium formate and certified reference materials of cocaine, ketamine, amphetamine, diazepam and tetrahydrocannabinol (THC) and cocaine‐D3 were obtained from Sigma‐Aldrich (Dorset, UK).

### 2.2 Sample Collection

A pub in a rural/residential area in the south of England was selected to collect dry swab samples from various surfaces within four locations. The exact name and location of the pub was agreed to be kept anonymous. The locations were chosen based on common touchpoints and areas known for illicit drug consumption: the men's and women's bathrooms, tables and the counter at the bar. Swabs were taken from the toilet cisterns, windowsills and toilet lids in both bathrooms which were made of porcelain; swabs were taken from various tables which were made from wood and swabs were taken across the 3‐m‐long bar which had a copper surface. Sterile cotton‐tipped swabs (Copan, Brescia, Italy) were used with a 5x5 cm template for each location, swabbing back and forth from the top to bottom corner while coating the entire swab tip with particulates from each surface. The swabs were then individually sealed and labelled in falcon tubes (Sterilin, Newport, UK). Five swabs were taken at each location over a three‐week period (*n* = 420).

### 2.3 Swab Extraction Procedure

The first three swabs of each location were analysed without any sample preparation. Optimisation of the extraction procedure by pipetting known concentrations of cocaine standards (1 and 0.5 ng/μL) onto 2 × 2 cm squares of fluorinated ethylene propylene (FEP) film (KOYOFEI, China) which were allowed to evaporate overnight. The dried samples were swabbed using the same cotton swabs used for sample collection and placed into individual falcon tubes. At both concentrations, samples were filled with 1 mL MeOH, similarly to the extraction of seized powder/tablets undertaken by Frinculescu et al. [22]. These were vortexed for 15 s and two tests were performed where the swab was pressed against the side of falcon tube to release analytes vs. using disposable tweezers to squeeze out the solvent. Squeezing out the solvent from the swab tips resulted in a higher extraction efficiency, with stronger signals produced from both concentrations. Optimisation was also carried out to be rapid, with the ability for similar methods to be used in mobile laboratories with minimal equipment. The falcon tubes containing the collected samples were unsealed and filled with 1 mL MeOH and re‐sealed and vortexed for 15 s. The swab was removed, and disposable tweezers (Avantor, Leuven, Belgium) were used to take the tip off the swab and squeeze out the remaining solution back into the falcon tube. A new set of tweezers was used for each swab to prevent cross contamination and a false positive result. The remaining solutions were pipetted into a 2 mL amber screw thread autosampler vial (Chromatography Direct, Chesire, UK) and the extracted swabs were discarded.

### 2.4 ASAP‐MS Analysis

The analysis of the swabs was performed on the Waters RADIAN ASAP Direct Mass Detector (Waters, Wilmslow, UK), which is a single quadrupole mass spectrometer. Liquid or solid samples are introduced to the ASAP source where the sample is desorbed by a stream of hot nitrogen gas (typically between 400°C–500°C), from the rod into the vapour phase, where it passes through a corona discharge region. The corona discharge is formed at the tip of a metal corona discharge needle held at 3 μA. Subsequent ionisation events can occur in‐source before reaching the inlet of the MS [23].

The first three swabs of each location were initially analysed using an untargeted method (acquisition method in full scan mode across a wide mass range) using the operating conditions as described in Table 1.

**TABLE 1:** Full scan parameters used with the Radian ASAP.

Mode	Positive
Drying gas	N_2_
Corona discharge current	3 μA
Source temperature	140°C
Probe temperature	450°C
Full scan mass range	*m/*z 50–500
Cone voltages	15, 25, 35, 55 V
Scan time	1 s

Samples were introduced to the source with borosilicate glass capillary rods (Fisher Scientific, Loughborough, UK). Prior to analysis, the glass rods were bulk cleaned by sonicating the tips in acetone for 15 min. The excess acetone was then discarded and the rods were dried in the oven at 75°C for 1 h. Sample introduction was achieved by rubbing the swab tip against all sides of the clean end of the glass rod. The swabs were returned and sealed into their original falcon tubes post analysis.

Once the swabs had been analysed directly, analytes were extracted off the swabs as described previously and analysed using the same untargeted method and a selected ion recording (SIR) targeted method. Conditions were optimised for largest signal intensity for the most abundant. Prior to analysis, the extracted samples were vortexed and loaded onto the glass rod by syringing 1 μL of the solution and allowing it to dry for 1 min. The parameters for the targeted analysis can be found in the Supporting Information (Table S1).

Additional blank extractions were made to compare their given signals with those of the analytes extracted from the swabs. A cleaned surface within the laboratory was used as a blank. The blank surface swabs were analysed with the untargeted and targeted method as previously described.

### 2.5 LC–MS Analysis

A targeted LC–MS analysis was carried out on a Waters SQ Detector single quadrupole mass spectrometer coupled to an ACQUITY UPLC H‐Class LC (Waters, Wilmslow, UK) with an electrospray ionisation (ESI) source. The extractions were diluted in HPLC grade water: acetonitrile (87:13). Mixed standards of 10, 20, 50, 80 and 100 pg/μL of cocaine, ketamine, THC, amphetamine and diazepam to assess the linearity of the instrument. The mixed standard of 100 pg/μL cocaine, ketamine, THC, amphetamine and diazepam from Sigma‐Aldrich (Dorset, UK) was created to estimate the approximate amount of analytes present. Blanks, standards and samples were ran in triplicate and a mean response was calculated. Blanks and standards were made up in solutions of 90:10 H_2_O:MeOH and the extracted samples further diluted with a dilution factor of 10 in H_2_O to 1 mL. The column used was an ACQUITY UPLC HSS T3 column (1.8 μm, 2.1 × 100 mm) (Waters, Wilmslow, UK) with a 5 μL inject volume and a flow rate of 0.4 mL/min. The column temperature was set at 50°C and the mobile phases were (A) water with 5 mM ammonium formate (pH 4) and (B) acetonitrile with 0.1% formic acid. Information on the gradient elution, MS conditions and Selected Ion Monitoring (SIM) parameters can be found in the Supporting Information (Tables S2–S4).

### 2.6 Data Analysis

#### 2.6.1 ASAP‐MS

Data analysis using the untargeted methods was carried out using MassLynx (version 4.2). Total ion chronograms (TIC) were extracted to view a full spectrum of all detected masses. Identification was based on direct interrogation of protonated molecules and at least two characteristic fragments as qualifier ions; an example of a positive detection can be found in the Supporting Information (Figure S1). Additionally, MassLynx was used to analyse the targeted data. All masses of interest were extracted using a *m/z* one window and the extracted ion chronogram was smoothed (mean smoothing, width = 3, number of smooths = 2) and integrated. The integrated peaks provided a peak area, which was compared to those of the blank signals. Positive identification required the peak area of the protonated molecule and at least two fragment ions to be at least three times greater than their corresponding blank. Systematic false positives were identified where the sample provided a positive response using ASAP‐MS and a negative response using LC–MS. The data acquired through the untargeted screening of the unextracted/extracted swabs was run through SpectralWorks AnalyzerPro XD (version 1.16) software for systematic false positives identified by the LC–MS data, knowns and unknowns which were manually verified. Library searching was applied using the SpectralWorks Radian ASAP Seized Drugs Library (version 4), applying a height and rejection threshold of 50% and 5% respectively to detect analytes.

#### 2.6.2 LC–MS

Data analysis was carried out using TargetLynx XS (version 4.1) application manager. Sample processing was used to identify and integrate samples based on their retention times and detection was only included where the retention time matched within a ±2 min retention window in the targeted analysis for the specific analyte referenced, alongside the positive detection of the mixed standard at 100 pg/μL.

#### 2.6.3 Statistical Evaluation of False Positive and Negative Rates

To evaluate the diagnostic performance of each ASAP‐MS analysis, a table was constructed using the confirmatory LC–MS data as the reference standard (true positive or negative detections). Method accuracy and diagnostic error rates were quantified by calculating screening sensitivity, specificity and the false negative rate (FNR) and the false positive rate (FPR). The FNR defined as the portion of LC–MS confirmed positive samples that failed to trigger a positive detection during the screening processes (FNR = FN/[TP + FN], where FN is the false negative and TP is the true positive). Sensitivity measures represent the inverse true positive rate (Sensitivity = TP/[TP + FN]). Specificity and false positive rates (FPR) were calculated relative to the total number of LC–MS confirmed negative samples. To assess the statistical precision and reliability, 95% confidence intervals (CI) were computed for all parameters using the Clopper–Pearson (exact binomial) method to account for sample size constraints and zero‐count frequencies. All statistical calculations and data transformations were made in Microsoft Excel and were converted into percentage values.

## 3 Results

### 3.1 LC–MS—Confirmatory Testing

A targeted LC–MS analysis was used to confirm the presence of the analytes of interest and to compare the RADIAN‐ASAP Detector for its ability to effectively screen illicit substances from a surface. The LC–MS data was used as the ‘true’ result for the data acquired from the RADIAN‐ASAP Detector and only cocaine and ketamine are reported here as they were the only target analytes detected.

Figure 1 presents a total of 320 positive detections with 250 of those being cocaine and 70 ketamine across the sampling period. Over half of the swabs were found to contain cocaine, with the most cocaine found in the men's bathroom followed by the women's. Ketamine also emerged in most of the swabs taken in both bathrooms while both analytes were found in the table and bar areas to a lesser extent. A large increase in ketamine detections was found using LC–MS that was not detected with ASAP‐MS, with 64 of the swabs containing both analytes. The analysis shows that Week 2 had the highest number of positive detections (117) followed by Weeks 3 and 1 (109, 95 detections).

**FIGURE 1:**
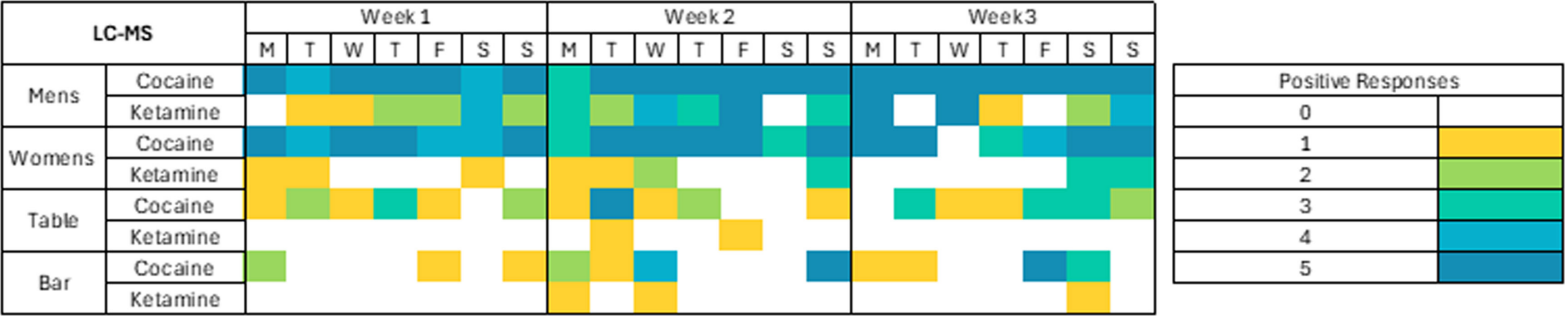
Heatmap of the number of positive detections for cocaine and ketamine in all four locations in sampling weeks 1–3 using a single‐quadrupole LC–MS system.

For the estimation of the amounts of each analyte present in the samples, the instrument was tested for its linearity using a 5‐point calibration curve for cocaine and ketamine.

The results displayed in Figure 2 display high coefficient of determination (*R* ^2^) values of over 99% for both analytes and demonstrates large linearity. Regression analysis was applied to both linear models and the output can be found in the Supporting Information in Tables S5–S6 and Figures S2–S3. The linear regression analysis of the calibration curve yielded *p*‐values of less than 0.05 (*p* < 0.05), demonstrating a statically linear relationship at a 95% confidence interval. As the aim of this research was to use LC–MS and a confirmatory technique, a single mixed component standard of 100 pg/μL with cocaine D‐3 as the internal standard was used alongside the analysis of the samples of their detection. Given the proven linearity of the instrument, a single point calibration using the 100 pg/μL mixed standard as used to estimate the amounts of analyte that were present in the samples. A plot of the standard was created to monitor its performance each time it was used in an analytical run and can be found in the Supporting Information in Figures S4–S5. From the 250 positive cocaine detections, 230 where detected within two orders of magnitude of the standard response and the remaining 20 exceeded two orders of magnitude, which were deemed as having a ‘high level’ of cocaine present and all the positive detections of ketamine where within the two orders of magnitude. All samples containing high levels of cocaine where those taken from the either the men's or women's bathroom, with the higher amount of samples containing high levels being recorded on Tuesday Week 3 in the men's bathroom.

**FIGURE 2:**
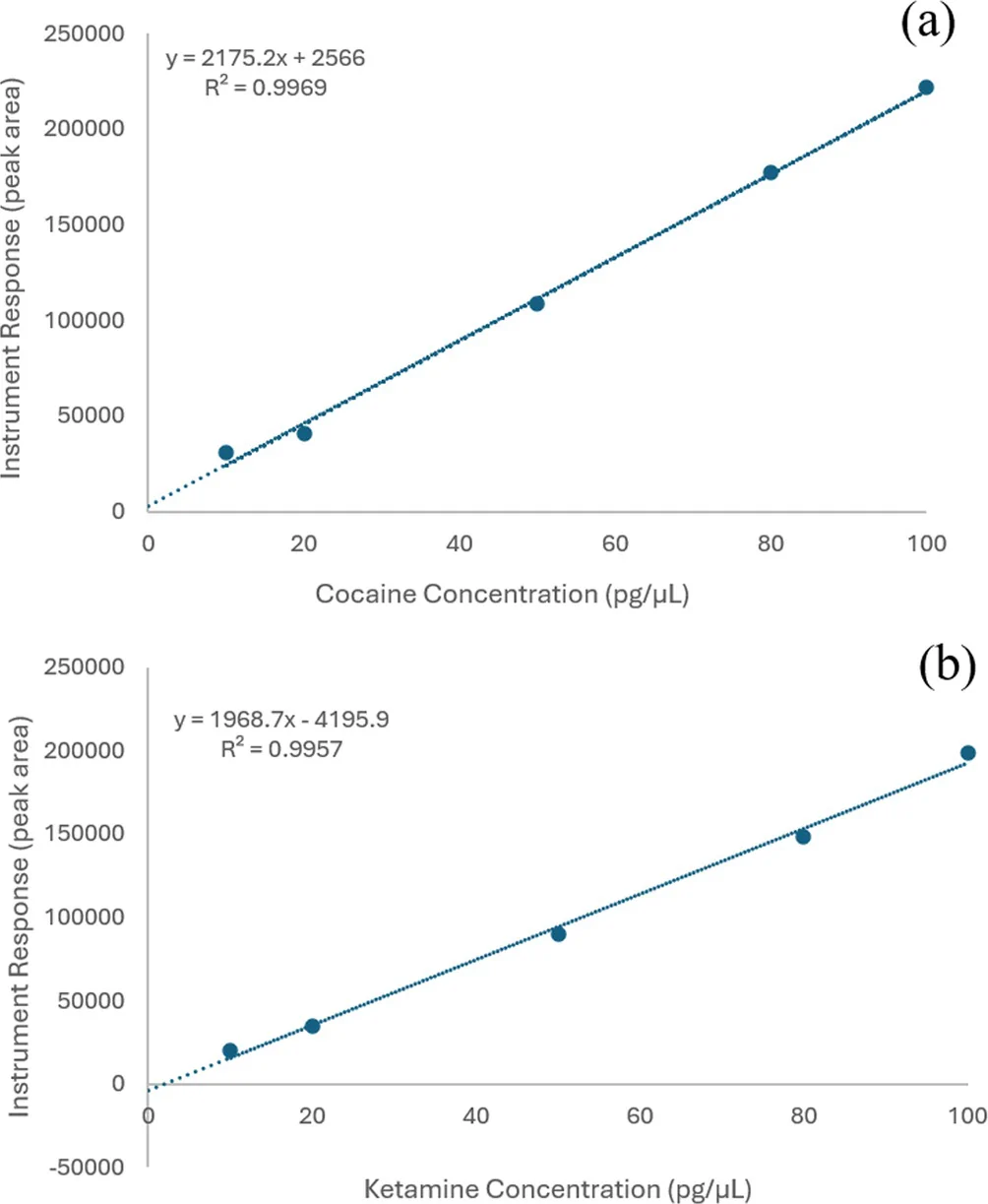
Calibration plots of 10, 20, 50, 80 and 100 pg/μL of (a) cocaine and (b) ketamine with the linear equations and *R* ^2^ values and an extrapolated regression line to reach the origin.

### 3.2 Screening With ASAP‐MS

#### 3.2.1 Untargeted Analysis With Swabs

Out of the five swabs, only three swabs were chosen to directly analyse. All analysis that involved solvent extractions were achieved with all five swabs. A full scan method produces a chronogram of any constituents present in a chosen *m/z* range and by directly transferring particulates from the swab onto the glass rod eliminates the need for sample preparation. False positives are indicated with a cross and were identified based on the LC–MS data.

The results in Figure 3 display that overall, there were 53 positive detections across the three‐week period with 47 of those being cocaine and six ketamine with three of those appearing as false positives. Week 2 displayed the most positive detections (23), followed by Weeks 1 and 3 (20, 10, respectively). However, cocaine appears to be the most prevalent analyte, particularly in the men's bathroom which contains most of the responses. Following this, the women's bathroom detected cocaine nine times across the sampling period as well a cocaine detection in the table areas in Week 1 and a detection of ketamine in Week 2. The first three swabs from the five were used to determine if transferring particulates from a swab would influence the confirmatory analysis, where ASAP‐MS would detect analytes where LC–MS did not. There were no instances where ASAP‐MS detected either cocaine or ketamine where LC–MS did not, displaying that despite particulate transfer, detection through confirmatory analysis would still be possible and enough of the original sample would still be present.

**FIGURE 3:**
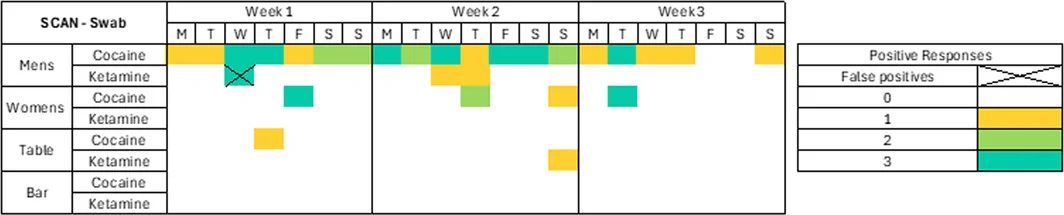
Heatmap of the number positive detections for cocaine and ketamine in all four locations in sampling Weeks 1–3 from rubbing the swab against a glass rod using an untargeted method.

#### 3.2.2 Untargeted Method With Solvent Extractions

All swabs were extracted in methanol and syringed onto a glass rod and analysed using the same untargeted method as previously described.

Figure 4 displays that across the 3 weeks there were a total of 97 positive detections with 89 of those containing cocaine and seven being ketamine (with the same three samples identified as false positives as previously mentioned). The overall detection of cocaine has doubled with solvent extracted samples compared to the direct analysis; however all five swabs were analysed here, rather than only three. This method displays Week 1 with the highest number of overall detections (37) followed by Weeks 2 and 3 (36, 21 respectively). Similarly to the untargeted analysis without sample preparation, we see a high number of positive detections for cocaine in the men's bathroom, where nearly all samples contain at least one positive detection. The untargeted analysis of the extracted swabs detected cocaine seven times more than the initial untargeted analysis for the men's bathroom from the first three swabs analysed. Despite this, there does appear to be instances where an analyte was detected with the initial untargeted analysis but missed in the untargeted analysis after being extracted. For example, Sunday Week 3 for the men's bathroom, cocaine was detected on one of the swabs when analysed directly; however it was not detected after extraction in untargeted analysis. With the addition of the remaining two swabs, the same trends appear to follow with more positive detections of cocaine in the women's bathroom.

**FIGURE 4:**
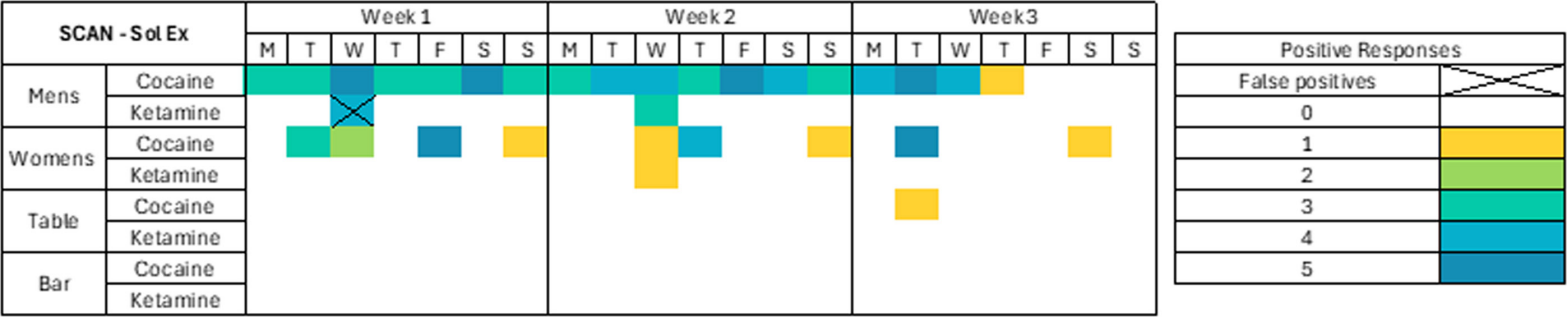
Heatmap of the number of positive detections for cocaine and ketamine in all four locations in sampling Weeks 1–3 from syringing 1 μL of the solvent extracted swabs with the untargeted method.

#### 3.2.3 Targeted Method With Solvent Extractions

A targeted analysis allows for the detection of specified *m/z* values which subsequently leads to increased sensitivity. A targeted method was used with the extracted swabs to assess and compare the RADIAN‐ASAP Detectors ability for screening using targeted vs. untargeted analysis.

With a targeted approach, the overall number of positive detections was 118 with 104 of those being cocaine and 14 being ketamine. However, using a targeted method presented an increased number of false positives with two being cocaine and six ketamine, which is further discussed in Section 4.3. Despite the false positives, a large increase of positive cocaine detections is evident as for ketamine at a lesser extent. As previously discussed, Week 1 displayed the highest number of positive detections followed by Weeks 2 and 3 and this pattern has continued with the targeted results. Additionally, the targeted analysis continued to display the men's bathroom with a high number of positive detections of cocaine however there was an increased number of cocaine detections found in the women's bathroom and cocaine and ketamine in the table and bar areas. Similarly to the extracted untargeted results, there were instances where the targeted analysis did not detect either illicit substance whereas the untargeted did.

#### 3.2.4 Lowest Concentrations Detected With ASAP‐MS

The instrument sensitivity was derived using the average response of three replicate runs of a known standard of cocaine and ketamine using the untargeted method. The peak‐to‐peak signal to noise (p:p *S/N*) was then determined to assess the limit of detection a S/N of 3:1 using the signals produced by the protonated molecular ions. This resulted in a LOD of 13 pg/μL and 0.14 ng/μL for cocaine and ketamine respectively. The semi‐quantification of the samples using LC–MS allowed for comparison with those detected with ASAP‐MS to estimate the lowest concentration detected with different modes of analysis to gain insight of the threshold of the instrument to detect analytes from complex sample matrices.

The lowest detected concentrations were above the calculated LOD which is expected as they were not pure standards. The lowest quantity of cocaine was detected at approximately 20 pg/μL across all three methods, representing the lowest concentration approximated by LC–MS. However, the targeted method, detected a greater number of samples with similar low concentrations compared to both untargeted analyses. With the untargeted method, cocaine was detected at two concentrations within the 20–30 pg/μL range, while the remaining detections were above 5.34 ng/μL, likely reflecting variability in analyte distribution or differences in swab‐to‐glass rod transfer efficiency. Ketamine was detected at low nanogram levels in the targeted method, in contrast to the untargeted approach, as indicated by its presence in both bar and table samples. Both untargeted analyses detected concentrations as low as of 3.40 ng/μL, with solvent‐extracted swabs producing a higher number of positive ketamine detections and the targeted method detecting significantly lower than the untargeted method for ketamine. However, these values approximate see what concentration ASAP‐MS can detect either analyte in complex mixtures rather than in a pure standard.

### 3.3 False Negative and Positive Rates

The diagnostic performance of each ASAP‐MS analysis was investigated against the confirmatory LC–MS analysis. Table 2 displays the FNR and FPR values for cocaine and ketamine with calculations on the sensitivity, method specificity and overall accuracy based on these values.

**TABLE 2:** FNR and FPR values for cocaine and ketamine in each ASAP‐MS analysis explored in this study based on the true positive and negative detections from the confirmatory LC–MS analysis.

	ASAP‐MS					
	Untargeted direct analysis		Untargeted solvent extraction		Targeted solvent extraction	
	Cocaine (%)	Ketamine (%)	Cocaine (%)	Ketamine (%)	Cocaine (%)	Ketamine (%)
FNR	72.84	95.12	64.80	94.29	59.51	88.57
FNR 95% CI lower limit	65.30	83.47	58.53	86.01	53.11	78.72
FNR 95% CI upper limit	79.52	99.40	70.71	98.42	65.69	94.93
Method sensitivity	27.16	4.88	35.20	5.71	40.49	11.43
Sensitivity 95% CI lower limit	20.48	0.60	29.29	1.58	34.31	5.07
Sensitivity 95% CI upper limit	34.70	16.53	41.47	13.99	46.89	21.28
FPR	0.00	1.42	0.00	0.86	1.73	1.71
FPR 95% lower limit	N/A	0.29	N/A	0.18	0.36	0.63
FPR 95% upper limit	N/A	1.54	N/A	0.89	0.25	3.21
Method specificity	65.30	83.47	58.53	86.01	53.11	78.72
Specificity 95% CI lower limit	95.98	95.90	97.85	97.52	95.02	96.31
Specificity 95% CI upper limit	N/A	99.71	N/A	99.82	99.64	99.37
Overall accuracy	53.17	83.33	61.43	83.57	64.29	83.81
Accuracy 95% CI lower limit	46.81	78.15	56.59	79.67	59.50	79.93
Accuracy 95% CI upper limit	59.46	87.72	66.11	86.99	68.87	87.20

The diagnostic performance of the ASAP‐MS screening methods varied significantly across analytes, methods and sample introduction workflows. For cocaine, moving from the untargeted direct analysis of the swabs to the untargeted solvent extraction and finally to the targeted solvent extraction, incrementally reduced the FNR from 72.84% (95% CI: 65.30%–79.52%) to 64.80% (95% CI: 58.53%–70.71%) and 59.51% (95% CI: 53.11%–65.69%), respectively. This shift correspondingly increased method sensitivity from 27.16% to 40.49%. A similar trend was observed for ketamine, where the high FNR of 95.12% (95% CI: 83.47%–99.40%) in the untargeted direct analysis of swabs was suppressed to 88.57% (95% CI: 78.72%–94.93%) using the targeted solvent extraction, elevating method sensitivity from 4.88% to 11.43%.

Although for cocaine, both untargeted methods maintained an optimal FPR of 0.00%, the introduction of a targeted solvent extraction matrix introduced a minor trade‐off, elevating the cocaine FPR to 1.73% (95% CI: 0.36%–0.25%). Across all three preparation modes, ketamine consistently exhibited an FPR below 1.75%. Overall diagnostic accuracy was highest when using targeted solvent extraction, reaching 64.29% (95% CI: 59.50%–68.87%) for cocaine and 83.81% (95% CI: 79.93%–87.20%) for ketamine. Despite these preparation enhancements, both analytes, particularly ketamine, retained exceptionally high false‐negative rates, indicating a persistent screening detection deficit across all evaluated workflows.

## 4 Discussion

### 4.1 Comparison of ASAP‐MS Analyses

Initial analysis was performed using an untargeted method to directly examine the swabs. This approach involved transferring particles directly to a glass rod, enabling rapid analysis with no sample preparation. The untargeted method used four cone voltages which created a multi‐list of fragments that were acquired simultaneously during data acquisition. This could gather information across a broad mass range of analytes and their characteristic fragment ions. From transferring analytes to a glass rod to data acquisition, the entire process took approximately 2 min. This allows for direct contact with the sample, with four mass spectrums generated in a rapid analysis time. This makes the method suitable for on‐site testing in scenarios where materials or equipment may be limited or challenging to transport and the method simplicity makes it accessible for use by non‐specialists. Despite the advantages, the fragility of the glass rods is challenging, as insufficient pressure applied to the rod may lead to poor particulate transfer and false negatives.

The untargeted analysis successfully detected most of the cocaine present on swabs collected from the men's bathroom; however it appeared to miss a significant portion of the cocaine later identified (by LC–MS) in the women's bathroom and it correctly identified ketamine in three swabs. Cotton swabs that were used in this study are commonly used to lift illicit trace residues, due to their high porosity and ability to trap solids. However, if the sampling surface is wet, the cotton swab can pull analytes deep into its core via capillary force [24]. Each sampling surface examined may have had some degree of moisture present, particularly the table/bar areas where there may have been a presence of spilt drinks or condensation from glasses. This may have resulted in analytes becoming trapped within the swab, rather than remain on the outside surface where they could be readily transferred. A majority of the ‘high levels’ of cocaine were detected from the men's bathroom from the LC–MS analysis which were all detected by the untargeted direct analysis of the swabs. These amounts could be large enough to saturate the swab and allow for particulates to remain on the outer surface to be transferred. Higher amounts of illicit substances are likely to be present in public bathrooms as such spaces are widely recognised as common sites for illicit drug use [25]. Such spaces offer a degree of privacy within public venues, contributing to their frequent utilisation for substance abuse and hence high levels of contamination. No ketamine detection resulted in ‘high levels’ however dry crystalline ketamine has also been found to have a poor transfer efficiency to glass materials through mechanical contact alone, with higher efficiencies shown when the swab has been wetted with solvent [26].

Using the same untargeted method with solvent extracted samples, releases the analyte trapped within the cotton fibres. Methanol was used as the extraction solvent and due to its rapid evaporation time, allowing for analysis in less than 1 min (when using a volume of 1 μL). The untargeted method applied to solvent‐extracted samples successfully detected cocaine that had initially been missed (e.g., in the women's bathroom on Saturday of Week 3) and ketamine in the men's bathroom (Wednesday of Week 2). With a higher number detection of analytes where direct swabbing had missed, it is likely that analytes were trapped within the swab or had adhesion forces that could not be overcome by mechanical contact alone. Although this method requires sample preparation, the process is minimal where 2–3 min is required per sample and uses portable, space‐efficient equipment suitable for on‐site applications.

There are two instances where ASAP‐MS analysis of solvent‐extracted samples failed to detect analytes that had been identified during the initial analysis (e.g., cocaine in the men's bathroom on Sunday of Week 3). This discrepancy could be attributed to either.

1. That substantial transfer occurred during the initial swabbing of the glass rod, allowing detection at that stage. Consequentially, when transferring particulates from the swab to the rod, a loss of material may have occurred, leaving less analyte available for subsequent extraction.
2. The use of 1 μL of methanol may have been insufficient to transfer enough analyte for positive detection. Although small volumes facilitate rapid drying, larger volumes could enhance analyte transfer and hence detection.

The use of an untargeted method, with solvent extracted samples or direct analysis, offers the advantage of rapid analysis while simultaneously detecting a broad range of analytes. However, the method of sample introduction may be limited for some analytes particularly for compounds at trace levels and if the sampling surface is wet. The direct transfer of analytes from a swab may be better suited to swabbing dry, bulk powders, or swabbing particles that are visible to the naked eye. Solvent extraction would be beneficial for surfaces that appear ‘clean’ or have any degree of moisture, particularly for analytes like ketamine which have strong bonds to cotton. Additionally, illicit substances may be localised to a specific area and the probability of effective transfer from swab to rod becomes less likely, but solvent extractions would eliminate this challenge. In the context of this study, public bathrooms are more likely to retain higher residual levels of substances on surfaces. These elevated amounts increase the likelihood of detection using an untargeted method. In contrast, surfaces such as tables and bar countertops, although potentially exposed to illicit substances, are more accessible to patrons and staff. As a result, they are subject to more frequent cleaning or may only contain trace amounts due to incidental contact, such as hand‐to‐surface contamination. Despite the increased number of positive ketamine detections produced from solvent extractions, many of them were still missed that were identified by LC–MS. Idoudi et al. suggest that the optimal solvent for dissolving ketamine hydrochloride (HCl), which is highly likely to be found in street samples rather than its freebase, is plain water where ketamine HCl is highly soluble [26]. Although methanol has a rapid drying time and typically covers a wide range of analytes, other solvents or mixes should be investigated for comparison of extraction efficiencies.

The targeted method with extracted swab samples detected the greatest number of samples that were positive for ketamine and more positive detections for cocaine and ketamine in the table and bar areas. With the increased number of detections also came the increased number of false positives. ASAP‐MS does not provide compound separation, resulting in complex spectra where matrix interferences and isobaric species can produce signals at the same *m/z* as the target analytes [21]. To understand potential interferences, procedural blanks (in addition to swab blanks) were prepared by swabbing clean laboratory surfaces. These serve to establish threshold signal levels however, they are not true environmental blanks (are likely different in chemical composition, cleanliness and contamination and therefore may not fully represent the matrix present in the samples). The benefit of having a targeted method would appear to be the increased number of detected analytes which is likely due to the higher sensitivity a targeted method possesses. This would be beneficial for samples containing trace amounts as well those that contain a high amount of interferences, i.e., drinks residue or cleaning products which could suppress the analytes signal. A method can be created that contains both an untargeted and targeted method, which would allow for targeted screening for compounds of interest as well as screening for new or unknown substances.

### 4.2 ASAP‐MS Screening vs. LC–MS Confirmatory Testing

LC–MS analysis revealed a large presence of cocaine and ketamine in bathroom areas, with lower levels detected on tables and bar surfaces. LC–MS offers high sensitivity and specificity through chromatographic separation, making it particularly effective for detecting trace levels of compounds even in chemically diverse or contaminated samples. This enhanced capability comes with trade‐offs, most notably extended run times and more complex sample preparation making it unsuitable for field analysis. Despite this, the targeted method detected the greatest number of illicit substances compared to the untargeted methods, likely due to its sensitivity and optimised conditions for each analyte of interest.

Although each ASAP‐MS analysis was able to detect either analyte, from the diagnostic performance there was an overwhelming number of false negative responses. The higher discrepancy between the LC–MS analysis vs. all the ASAP‐MS analyses performed was the detection of ketamine. As previously discussed, there could have been challenges with the transfer of analytes from the swab to the glass rod, solvent extraction procedures and sampling volumes. Many of the swabs containing ketamine also contained cocaine and once analytes are introduced to the source, there will be a competition of thermal desorption from the glass rod that occurs. As ASAP‐MS is not coupled with a separation technique, samples are vaporised sequentially based on their individual boiling points and thermal stability. Cocaine is characterised with a high volatility and lower boiling point with efficient desorption at temperatures between 150°C–220°C and prone to thermal degradation at temperatures exceeding 350°C–550°C [27]. Ketamine typically decomposes in the temperature range of 258°C–263°C and thermal desorption techniques have been used with temperatures of 250°C for 2–5 min to vaporize ketamine without destroying the molecule [28]. Cocaine has a significantly higher volatility compared to ketamine and would readily desorb off the glass rod and ketamine would remain where it desorbs at a much slower rate. As the data acquisition times were taken within seconds, there is a possibility that there was an insufficient amount of time for enough ketamine to desorb off the glass rod. All the ASAP‐MS methods had a fixed probe temperature of 450°C, which has been used in ASAP‐MS studies involving the analysis of bulk illicit powders/tablets [21, 22]. However, this temperature may be too high where trace amounts are present, as it could cause the analytes to rapidly decompose to the point where the absolute number of vapourised molecules is low and not saturate the corona discharge region. Cocaine was detected using ASAP‐MS at a higher rate compared to ketamine and exhibited estimates concentrations that either exceeded two orders of magnitude of the linear range or lied towards the higher end of the linear range or exceeded one order of magnitude of the LC–MS analysis. Most of these values were present in the men's bathroom across the sampling period and were detected by the solvent extracted samples in the untargeted/targeted analysis. There may have been enough cocaine for enough vapourised molecules to reach the source despite thermal decomposition. Conversely overall ketamine detection was detected within one order of magnitude of the linear range and may have resulted in almost complete thermal decomposition which could have resulted in the false negatives.

Once analytes are desorbed there is also a competition for ionisation. Cocaine had a higher gas‐phase proton affinity and stronger basicity compared to ketamine [29]. With cocaine being present in larger quantities compared to ketamine and its stronger ability to compete for available charges, it is likely that cocaine may have suppressed the signals produced by ketamine when both are present. This is reflected in the results of the untargeted analysis of direct and extracted swabs, where no samples that contained both analytes detected ketamine. Upon inspection of the data, cocaine signals were either strong and completely dominated the spectrum or still had the presence of the [M + H]^+^ ion with higher levels of noise and unknown masses (see Supporting Information Figure S6). Additionally, the findings from Table 3 show that cocaine can be approximately detected at a range that is three orders of magnitude lower than ketamine, which can affect the competition for ionisation. Some of these samples were later identified using the targeted analysis which monitored unique fragment ions for each analyte independently, despite suppression of the signals. Another variable would include other compounds present on the sampling surfaces the time of swabbing, resulting in additional competition for thermal desorption and ionisation. To overcome these limitations, the fixed probe temperature could be replaced with a temperature ramp. The ramp would allow for the temperature to slowly increase from 100°C to 600°C to stretch out the desorption timeline and would add an extra 30s for data acquisition. This would effectively create a degree of separation between analytes as it would allow for low‐boiling components to desorb sequentially before degradation could occur. This would result in molecules reaching the corona discharge region at different times, minimising their competition for charge and ion suppression with a simpler mass spectrum [30]. Additionally, samples that produce signals of an analyte that dominates the spectrum could be diluted to avoid saturation and ion suppression of analytes that are present at lower concentrations or with lower proton affinities.

**TABLE 3:** Approximate thresholds of cocaine and ketamine detection with ASAP‐MS using untargeted analysis of swabs and untargeted/targeted analysis of extracted swabs with ± relative standard deviation (RSD) values.

Method	Cocaine (pg/μL) ± RSD	Ketamine (ng/μL) ± RSD
Untargeted (SCAN)—swabs	20 ± 0.23	3.40 ± 1.0
Untargeted (SCAN)—extracted swabs	20 ± 0.23	3.40 ± 1.0
Targeted (SIR)—extracted swabs	20 ± 0.23	0.78 ± 0.85

Despite the limitations, the methods used in this study for ASAP‐MS were able to identify analytes of interest. Each method of analysis and sample introduction had its own advantages and limitations however, it allows for versatility depending on sample type and collection. Bulk or visible quantities of sample would be ideal for direct analysis and trace amounts should be extracted to release analytes trapped to the surface or deep within the swab. A targeted and untargeted analysis could be used together to discover unknowns and detect trace amounts of target analytes. Results from the LC–MS analysis revealed a much larger presence of cocaine and ketamine across the sampling period however, LC–MS possesses separating power that ASAP‐MS does not. However, the addition of a temperature ramp would likely increase the number of positive detections for cocaine and ketamine by ASAP‐MS and allow for less complex spectra and ion suppression. This would assist in detecting analytes in complex matrixes which could include mixtures containing more than two drugs, with low and high proton affinities and varying desorption efficiencies.

### 4.3 False Positives and Unknown Analytes

Across all ASAP‐MS methods, swabs 1–3 and 4 from Wednesday Week 1 in the men's bathroom were identified as systematic false positives for ketamine. The data acquired for these swabs were processed through AnalyzerPro XD with a library containing approximately 80 target analytes. The software identified the presence of procaine with a 97.2% confidence which is displayed in Figure 5.

**FIGURE 5:**
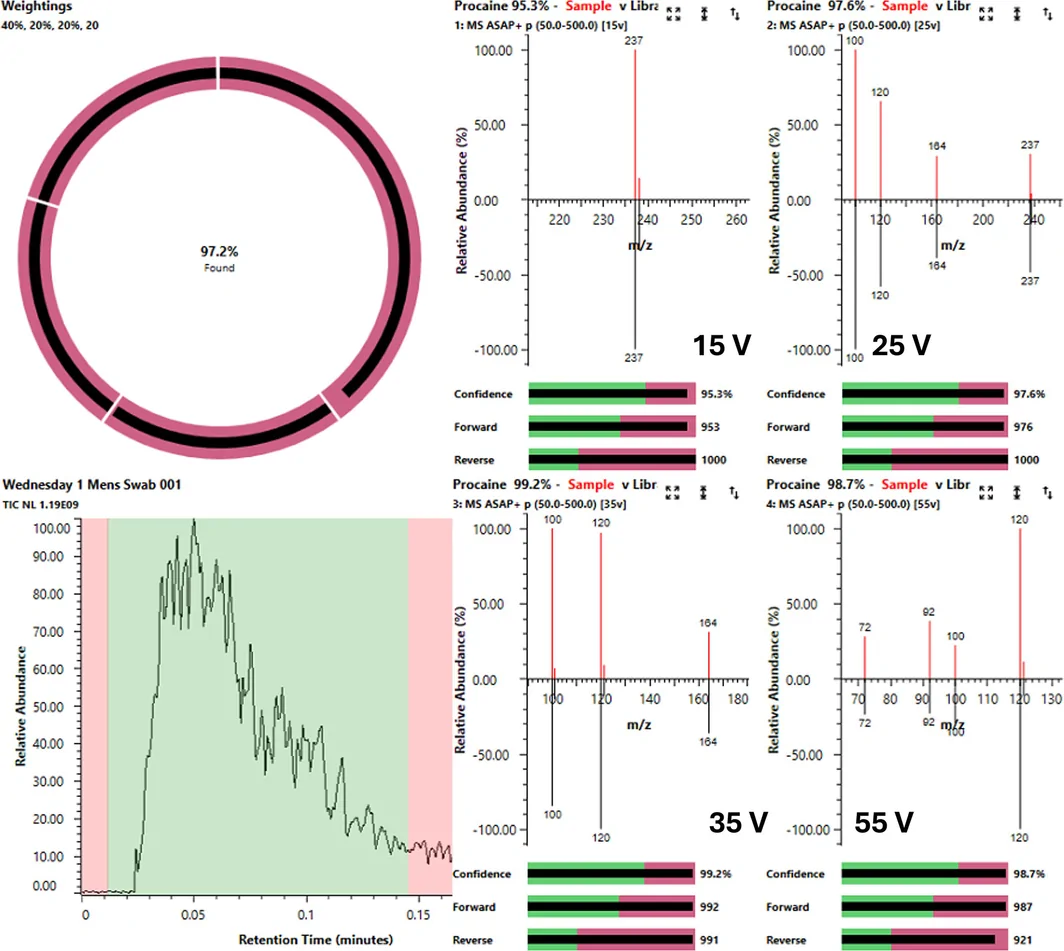
Results of men's bathroom swabs from Wednesday Week 1 using AnalyzerPro XD displaying the detection of procaine (protonated molecular ion *m/*z 237) and known fragments (*m/*z 164, 120, 100, 92 and 72) matched with those in the target library.

Procaine is occasionally used as a cutting agent for cocaine and found to reliably produce the [M + H]^+^ at *m/z* 237 under ambient ionisation [31]. Procaine's protonated ion is one mass unit away from ketamine (*m/z* 238), producing a false signal for *m/z* 238 may be due to a lack of resolution or from the isotopic addition of C^13^ which could be confirmed by further analysis with high‐resolution mass spectrometry. Figure 6 displays four spectra for procaine at the four different voltages within the method and the protonated ion and fragments are all present with confidence scores of 95%–99%, suggesting that it is highly likely that procaine is present in the sample. Overall, there were a total of 15 false positives identified across all three ASAP‐MS methods, most of which were identified by the targeted analysis that may be due to the procedural blanks and the systematic false positives were found to be procaine. Additionally, the software was able to identify unknown analytes that were not the focus of this study. This included additional procaine detections from swabs obtained in the men's and women's bathrooms as well as phenacetin and caffeine which have previously been detected in seized cocaine samples [32]. Two illicit substances were identified once which were temazepam and para‐methoxymethamphetamine (PMMA) which is often sold as ‘ecstasy’, see Supporting Information for lists of detected unknowns and their sample number/location (Tables S7–S8). Software such as AnalyzerPro XD can be integrated with data acquisition to provide a confidence score for the detection of analytes of interest and unknown analytes.

**FIGURE 6:**
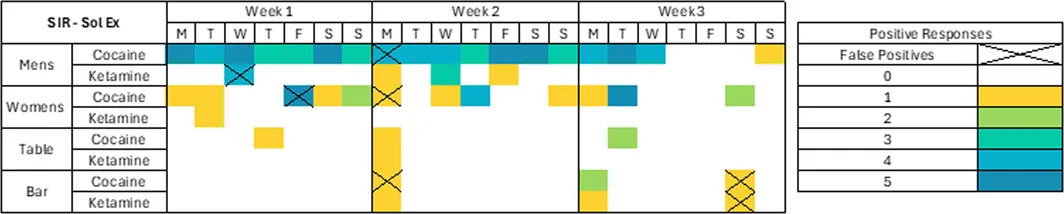
Heatmap of the number of positive detections for cocaine and ketamine in all four locations in sampling Weeks 1–3 from syringing 1 μL of the solvent extracted swabs with the targeted method.

## 5 Conclusion

The requirement for rapid and reliable screening techniques remains essential. This study assessed the utility of ASAP‐MS (using untargeted and targeted methods) alongside LC–MS for the detection of illicit drugs on surfaces within a public venue. ASAP‐MS offered the advantage of rapid, minimal‐preparation analysis. The untargeted method effectively detected high analyte concentrations, in the bathrooms which are typically less frequently cleaned environments, whereas the targeted method significantly enhanced sensitivity, particularly for ketamine. Solvent extraction further improved analyte recovery by liberating residues retained on swabs, though some inconsistencies were observed, likely due to variability in sample transfer and minimal extract volumes. Initial ASAP‐MS results suggested higher cocaine than ketamine prevalence over the three‐week sampling period; however, confirmatory LC–MS analysis revealed greater surface‐level ketamine presence. A total of 15 false positives were identified across the ASAP‐MS methods, with several ketamine signals later attributed to procaine which is a known cocaine adulterant. Due to the lack of resolution in ASAP‐MS, isobaric compounds such as procaine can interfere with accurate detection. Most false positives occurred with the targeted method, likely due to limitations in procedural blank accuracy. The integration of analytical software such as AnalyzerPro XD proved valuable in reducing false positives by providing compound–specific confidence scores and identifying potential cutting agents. Despite limitations, ASAP‐MS shows strong potential for rapid, cost effective and easy to operate on‐site screening. The untargeted analysis of swabs that allows for zero sample preparation may be useful in circumstances where large quantities of unknown substances are present, i.e., pills or bags of powder. For smaller quantities a quick extraction procedure can assist in detecting a higher quantity of samples using an untargeted method. The targeted analysis with solvent extracted samples shows strong potential as a rapid, on‐site screening tool for surface–level drug detection in complex environments. Additionally, a targeted approach may be beneficial for analytes of interest with method optimisation. The main requirement for optimisation appears to be the additional of a temperature ramp opposed to a fixed probe temperature. This would allow for sequential desorption of analytes with lower volatility and separation of compounds as they'll reach the source at different times. As a result, spectra would be less complex and minimises competition for thermal desorption and ionisation. Further optimization of sample volumes and matrix matched controls and additional data‐analysis software tools is recommended to enhance detection reliability and minimise false positives in future applications.

## Author Contributions

**Andriana Michailidis:** formal analysis; investigation (lead); methodology; visualisation; data curation; writing – original draft preparation. **Simone Mathias:** formal analysis; investigation (supporting); methodology; visualisation; writing – review and editing and original draft preparation. **Eloise Edgar‐Gibson:** resources. **David Douce:** formal analysis; resources; writing – review and editing. **Victoria Starkie:** resources; writing – review and editing. **Melanie Bailey:** writing – review and editing. **Patrick Sears:** conceptualisation; supervision; writing and review and editing.

## Supporting information


**Table S1:** Targeted analysis (SIR) parameters on the Waters Radian ASAP for protonated molecules and characteristic fragments.
**Table S2:** Gradient elution of mobile phase used in LC–MS analysis.
**Table S3:** MS conditions used in the LC–MS analysis.
**Table S4:** SIM method parameters and retention times for each target analyte in the LC–MS analysis.
**Table S5:**: Regression analysis results for cocaine for instrument response being a function of analyte concentration.
**Table S6:** Regression analysis results for ketamine for instrument response being a function of analyte concentration.
**Table S7:** List of unknown analytes detected using AnalyzerPro XD from the data acquired from the untargeted screening of the direct analysis of swabs.
**Table S8:** List of unknown analytes detected using AnalyzerPro XD from the data acquired from the untargeted screening of the analysis extracted swabs.
**Figure S1:** Spectra of the first swab of the men's bathroom Week 1. The spectra depicts a positive detection of cocaine using the untargeted method and analysing the swabs by rubbing them against a glass rod. The pronated molecular ion (*m/z* 304) can be seen at 15 V and characteristic fragments (*m/z* 182, 150, 105, 82 and 72) at 25, 35 and 55 V.
**Figure S2:** Standardised residuals vs. concentration plot for cocaine from the linear regression analysis. No unusual observations occurred for the fitted linear model as all standardised residual values are within < ±2. All experimental data and related predications are therefore within statical acceptable limits.
**Figure S3:** Standardised residuals vs. concentration plot for ketamine from the linear regression analysis.
**Figure S4:** Plot of monitoring 100 pg/μL cocaine response on each day an analytical run was done with cocaine‐D3 as the internal standard. Plot depicts the average response (x̄) and standard deviations (SD) as the error bars.
**Figure S5:** Plot of monitoring 100 pg/μL ketamine response on each day an analytical run was done with cocaine‐D3 as the internal standard. Plot depicts the average response (x̄) and standard deviations (SD) as the error bars.
**Figure S6:** Spectra of extracted swab using the untargeted method of swab 1 (top) and swab 2 (bottom) taken from the men's bathroom on Wednesday of sampling Week 2 in the lowest cone voltage (15 V). The top spectra depicts cocaine's protonated molecular ion *m/z* 304 with the highest intensity but surrounded by noise from background contamination or many compounds in the sample matrix. The bottom spectra clearly displays *m/z* 304 with a strong intensitiy and ion suppression of other constituents.

## Data Availability

The data that support the findings of this study are openly available in [https://zenodo.org/] at [DOI: 10.5281/zenodo.18306943].
