# Supplementary material for: Assessing the use of ASAP‐MS for Screening a UK Pub for Illicit Drug Substances
Source: J Mass Spectrom. 2026 Oct 12;61(11):e70121. doi: 10.1002/jms.70121 (PMC13659026; doi:10.1002/jms.70121)
Supplement: Supplementary file 1 [file JMS-61-e70121-s001.docx]

Supporting Information for

Screening and Confirmation Testing of Drugs detected within a UK Public House

**Authors:** Andriana Michailidis^1†^, Simone Mathias^1†^, Eloise Edgar-Gibson^1^, David Douce^2^, Victoria Starkie^2^, Melanie Bailey^3^ and Patrick Sears^1*^

**Affiliations:** ^1^ School of Chemistry and Chemical Engineering, University of Surrey, Guildford GU2 7XH, UK ^2^ Waters Corporation, Stamford Avenue, Wilmslow SK9 4AX, UK, ^3^ The Department of Infectious Diseases, King’s College London, London SE1 1UL

**Corresponding author:** [p.sears@surrey.ac.uk](mailto:p.sears@surrey.ac.uk)

^†^Joint first author

**Table S1:** Targeted analysis (SIR) parameters on the Waters Radian ASAP for protonated molecules and characteristic fragments.

| Compound Name | *m/z* | Cone Voltage (V) |
| --- | --- | --- |
| Amphetamine | 136 | 5 |
| Amphetamine Fragment 1 | 119 | 5 |
| Amphetamine Fragment 2 | 91 | 5 |
| Cocaine | 304 | 15 |
| Cocaine Fragment 1 | 182 | 35 |
| Cocaine Fragment 2 | 105 | 45 |
| Cocaine Fragment 3 | 82 | 40 |
| Cocaine Fragment 4 | 77 | 45 |
| Diazepam | 285 | 5 |
| Diazepam Fragment 1 | 257 | 35 |
| Diazepam Fragment 2 | 222 | 50 |
| Diazepam Fragment 3 | 154 | 50 |
| Ketamine | 238 | 15 |
| Ketamine Fragment 1 | 220 | 35 |
| Ketamine Fragment 2 | 179 | 35 |
| Ketamine Isotope | 240 | 15 |
| THC | 315 | 15 |
| THC Fragment 1 | 259 | 35 |
| THC Fragment 2 | 235 | 35 |
| THC Fragment 3 | 193 | 35 |

**Table S2:** Gradient elution of mobile phase used in LC-MS analysis.

| Time | A% | B% |
| --- | --- | --- |
| 0.00 | 87.0 | 13.0 |
| 0.50 | 87.0 | 13.0 |
| 7.00 | 50.0 | 50.0 |
| 7.75 | 5.0 | 95.0 |
| 11.25 | 5.0 | 95.0 |
| 11.50 | 87.0 | 13.0 |
| 14.00 | 87.0 | 13.0 |

**Table S3:** MS conditions used in the LC-MS analysis

| Mode | Positive |
| --- | --- |
| Capillary Voltage | 3 kV |
| Desolvation Gas | N_2_ |
| Desolvation Temperature | 400 °C |
| Desolvation Gas Flow Rate | 800 L/Hr |
| Source Temperature | 150 °C |
| Collision Gas | Argon |
| Cone Gas Flow Rate | 20 L/Hr |

**Table S4:** SIM method parameters and retention times for each target analyte in the LC-MS analysis.

| Compound | *m/z* | Cone Voltage (V) | Retention Time (mins) |
| --- | --- | --- | --- |
| Amphetamine | 136 | 15 | 1.87 |
| Ketamine | 238 | 30 | 3.09 |
| Diazepam | 285 | 50 | 4.16 |
| Cocaine | 304 | 45 | 8.41 |
| THC | 315 | 40 | 11.92 |

**Figure S1:** Spectra of the first swab of the men’s bathroom Week 1. The spectra depicts a positive detection of cocaine using the untargeted method and analysing the swabs by rubbing them against a glass rod. The pronated molecular ion (*m/z* 304) can be seen at 15 V and characteristic fragments (*m/z* 182, 150, 105, 82 & 72) at 25, 35 & 55 V.

**Table S5**: Regression analysis results for cocaine for instrument response being a function of analyte concentration

The *p* value is < the significance level and therefore rejects the null hypothesis of instrument response not being a function of analyte concentration at the 5% significance level. There appears to be sufficient evidence to indicate that the estimated linear equation of instrument response as a function of analyte concentration is statically valid (*p* < 0.05).

| Cocaine Concentration (pg/µL) | Standardised Residual |
| --- | --- |
| 24318 | 1.41171 |
| 46070 | -1.2241 |
| 111326 | -0.621116 |
| 176582 | 0.0965029 |
| 220086 | 0.337005 |

**Figure S2:** Standardised residuals vs. concentration plot for cocaine from the linear regression analysis.

No unusual observations occurred for the fitted linear model as all standardised residual values are within < ±2. All experimental data and related predications are therefore within statical acceptable limits.

**Table S6**: Regression analysis results for ketamine for instrument response being a function of analyte concentration.

The *p* value is < the significance level and therefore rejects the null hypothesis of instrument response not being a function of analyte concentration at the 5% significance level. There appears to be sufficient evidence to indicate that the estimated linear equation of instrument response as a function of analyte concentration is statically valid (*p* < 0.05).

| Ketamine Concentration (pg/µL) | Standardised Residual |
| --- | --- |
| 12.2709 | -0.904065 |
| 19.8573 | 0.0568294 |
| 47.6963 | 0.917144 |
| 77.3733 | 1.04575 |
| 102.802 | -1.11566 |

**Figure S3:** Standardised residuals vs. concentration plot for ketamine from the linear regression analysis.

No unusual observations occurred for the fitted linear model as all standardised residual values are within < ±2. All experimental data and related predications are therefore within statical acceptable limits.

|  | Cocaine Peak Area Ratio | Average | Upper Limits (x̄ + SD) | Lower limits (x̄ - SD) | Upper Limits (x̄ + 2*SD) | Lower Limits (x̄ - 2*SD) |
| --- | --- | --- | --- | --- | --- | --- |
| 1 | 3.90003 | 3.72773 | 3.86997 | 3.58549 | 4.01221 | 3.44325 |
| 2 | 3.63744 | 3.72773 | 3.86997 | 3.58549 | 4.01221 | 3.44325 |
| 3 | 3.74119 | 3.72773 | 3.86997 | 3.58549 | 4.01221 | 3.44325 |
| 4 | 3.66308 | 3.72773 | 3.86997 | 3.58549 | 4.01221 | 3.44325 |
| 5 | 3.70169 | 3.72773 | 3.86997 | 3.58549 | 4.01221 | 3.44325 |
| 6 | 3.59964 | 3.72773 | 3.86997 | 3.58549 | 4.01221 | 3.44325 |
| 7 | 3.7754 | 3.72773 | 3.86997 | 3.58549 | 4.01221 | 3.44325 |
| 8 | 3.57738 | 3.72773 | 3.86997 | 3.58549 | 4.01221 | 3.44325 |
| 9 | 3.76072 | 3.72773 | 3.86997 | 3.58549 | 4.01221 | 3.44325 |
| 10 | 3.63095 | 3.72773 | 3.86997 | 3.58549 | 4.01221 | 3.44325 |
| 11 | 3.87884 | 3.72773 | 3.86997 | 3.58549 | 4.01221 | 3.44325 |
| 12 | 4.05654 | 3.72773 | 3.86997 | 3.58549 | 4.01221 | 3.44325 |

**Figure S4:** Plot of monitoring 100 pg/µL cocaine response on each day an analytical run was done with cocaine-D3 as the internal standard. Plot depicts the average response (x̄) and standard deviations (SD) as the error bars.

|  | Ketamine Peak Area | Avergae | Upper Limits (x̄ + SD) | Lower Limits (x̄ - SD) | Upper Limits (x̄ + 2*SD) | Lower Limits (x̄ - 2*SD) |
| --- | --- | --- | --- | --- | --- | --- |
| 1 | 4.40001 | 4.35874 | 4.69882 | 4.01867 | 5.03889 | 3.67859 |
| 2 | 4.1133 | 4.35874 | 4.69882 | 4.01867 | 5.03889 | 3.67859 |
| 3 | 4.08174 | 4.35874 | 4.69882 | 4.01867 | 5.03889 | 3.67859 |
| 4 | 4.19252 | 4.35874 | 4.69882 | 4.01867 | 5.03889 | 3.67859 |
| 5 | 4.79174 | 4.35874 | 4.69882 | 4.01867 | 5.03889 | 3.67859 |
| 6 | 4.54169 | 4.35874 | 4.69882 | 4.01867 | 5.03889 | 3.67859 |
| 7 | 4.94497 | 4.35874 | 4.69882 | 4.01867 | 5.03889 | 3.67859 |
| 8 | 4.26553 | 4.35874 | 4.69882 | 4.01867 | 5.03889 | 3.67859 |
| 9 | 4.23473 | 4.35874 | 4.69882 | 4.01867 | 5.03889 | 3.67859 |
| 10 | 3.78904 | 4.35874 | 4.69882 | 4.01867 | 5.03889 | 3.67859 |
| 11 | 4.187 | 4.35874 | 4.69882 | 4.01867 | 5.03889 | 3.67859 |
| 12 | 4.76261 | 4.35874 | 4.69882 | 4.01867 | 5.03889 | 3.67859 |

**Figure S5:** Plot of monitoring 100 pg/µL ketamine response on each day an analytical run was done with cocaine-D3 as the internal standard. Plot depicts the average response (x̄) and standard deviations (SD) as the error bars.

**Figure S6:** Spectra of extracted swab using the untargeted method of swab 1 (top) and swab 2 (bottom) taken from the men’s bathroom on Wednesday of sampling week 2 in the lowest cone voltage (15V). The top spectra depicts cocaine’s protonated molecular ion *m/z* 304 with the highest intensity but surrounded by noise from background contamination or many compounds in the sample matrix. The bottom spectra clearly displays *m/z* 304 with a strong intensitiy and ion suppression of other constituents.

**Table S7:** List of unknown analytes detected using AnalyzerPro® XD from the data acquired from the untargeted screening of the direct analysis of swabs.

| Untargeted Analysis of Swabs | | |
| --- | --- | --- |
| Day and Week no., Location, Swab no. | Unknown Identified | Known Identified |
| Monday 2 Men’s 02 | Phenacetin | Cocaine |
| Wednesday 1 Men’s 01 | Procaine | Cocaine |
| Wednesday 1 Men’s 02 | Procaine | Cocaine |
| Wednesday 1 Men’s 03 | Procaine | Cocaine |
| Wednesday 3 Men’s 02 | Phenacetin | Cocaine |
| Thursday 1 Men’s 01 | Procaine | Cocaine |
| Thursday 1 Men’s 02 | Procaine | Cocaine |

**Table S8:** List of unknown analytes detected using AnalyzerPro® XD from the data acquired from the untargeted screening of the analysis extracted swabs.

| Untargeted Analysis of Extracted Swabs | | |
| --- | --- | --- |
| Day & Week no., Location, Swab no. | Unknown Identified | Known Identified |
| Monday 2 Men’s 01 | Phenacetin | Cocaine |
| Monday 2 Men’s 02 | Phenacetin | Cocaine |
| Monday 2 Men’s 03 | Phenacetin | Cocaine |
| Monday 2 Men’s 05 | Temazepam |  |
| Monday 3 Men’s 03 | Phenacetin | Cocaine |
| Wednesday 1 Men’s 01 | Procaine | Cocaine |
| Wednesday 1 Men’s 02 | Procaine | Cocaine |
| Wednesday 1 Men’s 03 | Procaine | Cocaine |
| Wednesday 1 Men’s 05 | Procaine | Cocaine |
| Wednesday 2 Men’s 01 | Phenacetin | Cocaine, Ketamine |
| Wednesday 2 Men’s 03 | Phenacetin | Cocaine, Ketamine |
| Wednesday 3 Women’s 05 | PMMA | Cocaine, Ketamine |
| Thursday 1 Men’s 01 | Procaine | Cocaine |
| Thursday 1 Men’s 02 | Procaine | Cocaine |
| Sunday 2 Men’s 03 | Caffeine | Cocaine |
| Sunday 2 Women’s 04 | Caffeine | Cocaine |
